# Effects of FGF21 overexpression in osteoporosis and bone mineral density: a two-sample, mediating Mendelian analysis

**DOI:** 10.3389/fendo.2024.1439255

**Published:** 2024-09-03

**Authors:** Jingjing Liu, Jun Jiang, Yunjia Li, Qiaojun Chen, Ting Yang, Yanfa Lei, Zewei He, Xiaowei Wang, Qiang Na, Changtao Lao, Xinlei Luo, Lirong Yang, Zhengchang Yang

**Affiliations:** ^1^ Department of Spinal Surgery, Southern Central Hospital of Yunnan Province, Honghe, China; ^2^ Department of Spinal Surgery, The Sixth Affiliated Hospital of Kunming Medical University, Yuxi, China; ^3^ Department of Oncology, Southern Central Hospital of Yunnan Province, Honghe, China

**Keywords:** fibroblast growth factor 21, osteoporosis, GWAS, Mendelian randomization, bone mineral density

## Abstract

**Objective:**

Fibroblast growth factor 21 (FGF21) is a secreted protein that regulates body metabolism. In recent years, many observational studies have found that FGF21 is closely related to bone mineral density and osteoporosis, but the causal relationship between them is still unclear. Therefore, this study used two-sample, mediated Mendelian randomization (MR) analysis to explore the causal relationship between FGF21 and osteoporosis and bone mineral density.

**Methods:**

We conducted a two-sample, mediator MR Analysis using genetic data from publicly available genome-wide association studies (GWAS) that included genetic variants in the inflammatory cytokine FGF21, and Total body bone mineral density, Heel bone mineral density, Forearm bone mineral density, Femoral neck bone mineral density, osteoporosis. The main analysis method used was inverse variance weighting (IVW) to investigate the causal relationship between exposure and outcome. In addition, weighted median, simple median method, weighted median method and MR-Egger regression were used to supplement the explanation, and sensitivity analysis was performed to evaluate the reliability of the results.

**Results:**

MR Results showed that FGF21 overexpression reduced bone mineral density: Total body bone mineral density (OR=0.920, 95%CI: 0.876-0.966), P=0.001), Heel bone mineral density (OR=0.971, 95%CI (0.949-0.993); P=0.01), Forearm bone mineral density (OR=0.882, 95%CI(0.799-0.973); P=0.012), Femoral neck bone mineral density (OR=0.952, 95%CI(0.908-0.998), P=0.039); In addition, it also increased the risk of osteoporosis (OR=1.003, 95%CI (1.001-1.005), P=0.004). Sensitivity analysis supported the reliability of these results. The effect of FGF21 overexpression on osteoporosis may be mediated by type 2 diabetes mellitus and basal metabolic rate, with mediating effects of 14.96% and 12.21%, respectively.

**Conclusions:**

Our study suggests that the overexpression of FGF21 may lead to a decrease in bone mineral density and increase the risk of osteoporosis, and the effect of FGF21 on osteoporosis may be mediated through type 2 diabetes and basal metabolic rate. This study can provide a reference for analyzing the potential mechanism of osteoporosis and is of great significance for the prevention and treatment of osteoporosis.

## 1 Introduction

Osteoporosis is a disease characterized by low bone mass, deterioration of bone tissue and destruction of bone microarchitecture, which can lead to impaired bone strength and increased risk of fracture (1). As a chronic disease of bone metabolism, it is the result of an imbalance between osteoblasts and osteoclasts (2, 3) and is ranked as one of the most serious diseases affecting health, being the second most common disease after heart disease. With the change of diet and lifestyle, the incidence of osteoporosis is increasing year by year, showing the characteristics of more and more young people. According to surveys, the prevalence of osteoporosis in adults over the age of 12 in the United States can reach 6.50%, which brings a huge economic and health burden to the affected population (4). It is expected that the related cost of osteoporosis will increase to 25.3 billion US dollars by 2025 (5). Fracture is an associated clinical complication of osteoporosis. The most common fractures are vertebrae (spine), proximal femur (hip), and distal forearm (wrist), which are also one of the main factors contributing to the increased risk of death in the affected population (6). The formation and development of osteoporosis are related to many factors, such as estrogen deficiency, age, and chemical agents. In addition, inflammation, oxidative stress and other factors can also accelerate the progression of osteoporosis (7, 8). Various signaling pathways, such as RANK/RANKL/OPG, Wnt/β-catenin, and estrogen signaling pathways, are also critical for regulating osteoclast and osteoblast activity (9). At present, osteoporosis has been highly valued and extensively studied by the society, and its treatment is mainly carried out for osteogenesis and osteoclast, such as vitamin D, diphosphonate, and selective estrogen receptor modulators (SERMs) (10). Cutting off the pathogenic factors and pathways of osteoporosis is also an effective way to prevent and treat osteoporosis, and there are many studies to explore this issue.

Fibroblast growth factor 21 is a secreted protein that regulates the metabolism of the body, but does not have the activity of promoting fibroblast growth and does not specifically bind to heparin (11). It can play a role in various tissues and organs such as brain, heart, skeletal muscle, kidney and intestine (12). FGF21 is upregulated in some tissues when subjected to different stimuli. In different organs, FGF21 expression is mainly induced by fasting, ketogenic and high-carbohydrate diets, free fatty acids, nuclear receptor agonists and other factors. In humans and animals, fasting strongly induces FGF21 expression. As an endocrine hormone, FGF21 acts on various organs mainly through FGF receptors. In liver, the physiological effect of FGF21 is mainly manifested in promoting fatty acid oxidation. In the muscles and pancreas, it increases insulin sensitivity and promotes glucose uptake. Its regulatory role in the brain increases energy expenditure for appetite suppression. In WAT, FGF21 is an effective inducer of mitochondrial brown fat uncoupling protein 1 (UCP1), which stimulates glucose uptake in WAT and BAT while promoting lipolysis and reducing body weight. Recent studies have confirmed that FGF21 may become one of the effective targets for the prevention and rehabilitation of metabolic diseases, and is widely used in the prevention and rehabilitation of metabolic diseases such as liver lipid, glucose and lipid metabolism. With the deepening of the research on FGF21, its role has been continuously excavated. Some scholars have found that FGF21 is closely related to bone mineral density and osteoporosis. Although observational studies have proved this view, the causal relationship between them is still unclear. Therefore, this study used two-sample, mediated Mendelian randomization (MR) analysis to explore the causal relationship between FGF21 and bone mineral density and osteoporosis, so as to provide reference for the prevention and treatment of osteoporosis.

## 2 Methods

### 2.1 Study design

In this study, Total body bone mineral density, Heel bone mineral density, Forearm bone mineral density, Femoral neck bone mineral density, osteoporosis as outcome variable, Single nucleotide polymorphisms (SNPs) significantly associated with FGF21 were selected as instrumental variables, and the causal relationship between exposure and outcome was analyzed by two-sample, mediated Mendelian randomization analysis. In order to verify the reliability of the results, heterogeneity test, pleiotropy analysis and sensitivity analysis were used to exclude the bias of the results. This study met the following three key assumptions: ① Strong correlations between instrumental variables and exposure factors; ② The instrumental variables were not correlated with any confounding factors associated with the outcome variables of exposure factors; ③ Instrumental variables can only affect outcomes through their association with exposure factors.

### 2.2 Data source

Analyses of MR Utilized publicly available GWAS summary data.FGF21 genetic data from the study of Zhao, including 14743 samples, 12957980 SNPs (13). Genetic data on osteoporosis were obtained from the UK Biobank with a sample size of 462,933 including 7547 cases and 455,386 controls. Through the IEU database (https://gwas.mrcieu.ac.uk/) to obtain the Total body bone mineral density (56284 samples, 16162733 SNPs), Heel bone mineral density (142,487 samples, 16,959,184 SNPs), Forearm bone mineral density (8143 samples, 9,955,366 SNPs),Femoral neck bone mineral density (32735 samples, 10586900 SNPs) four bone mineral density data were used as outcome variables. In order to avoid confounding factors due to racial differences, the genetic background of the study population was selected as European ethnicity.

### 2.3 Instrumental variable

We selected single nucleotide polymorphisms (SNPs) with strong and independent associations with exposure as instrumental variables (IV), and SNPs with P < 5×10^-8^ as outcome variables. However, due to the limited number of SNPs with FGF21 as exposure, We adopted a less stringent threshold (5×10^-6^) to capture more SNPs (14). The r^2^ threshold was set as 0.001 and 10000 kb to exclude linkage disequilibrium (LD), and the selected SNPs met F > 10. Studies show that eating and physical activity levels, body weight, BMI, gender is a risk factor for osteoporosis, in order to avoid confounding factors influence on the result, we identify and eliminate the SNPS associated with potential confounding factors.

### 2.4 Statistical analysis

Inverse variance weighting (IVW) was used as the main analysis method to assess the causal relationship between exposure factors and outcomes. weighted median, simple median method, weighted median method and MR-Egger regression were used to evaluate the reliability of IVW results, P<0. 05 was considered statistically significant. An F-value of >10 means that there is no weak instrumental variable bias. In this study, IVW method and MR-Egger method were used for heterogeneity test. When P>0. 05, there was no heterogeneity between SNPs. Sensitivity analysis was performed by Leave-one-out method and the results were visualized. Single SNP was excluded to observe whether it had an impact on the final results. The analysis of pleiotropy was performed by MR-PRESSO method. When P>0. 05, it was indicated that there was no pleiotropy.

### 2.5 Mediation Mendelian analysis

A two-step MR Analysis was performed to determine the mediating effect of type 2 diabetes and basal metabolic rate on the relationship between FGF21 and osteoporosis. We obtained data on type 2 diabetes (655,666 samples, 5030,727 SNPs) and basal metabolic rate (454,874 samples, 9851867 SNPs) as mediators through the IEU database. The proportion of type 2 diabetes and basal metabolic rate in the total effect was estimated by dividing the indirect effect by the total effect (β1 × β2/β3), where β1 represents the effect of FGF21 on type 2 diabetes and basal metabolic rate, β2 represents the effect of type 2 diabetes and basal metabolic rate on osteoporosis, and β3 represents the effect of FGF21 on osteoporosis.

## 3 Results

### 3.1 Instrumental variable

After removing IVs with linkage disequilibrium from FGF21 genetic data, a total of 24 SNPs were included in this study, and the basic information of SNPs is shown in **Table 1**. The corresponding F values of single SNP in this study were all >10, there was no weak instrumental variable bias, and the results were reliable.

**Table 1:** Basic information on SNPs of FGF21.

SNPs	CHR	Position	EA	OA	β	EAF	P
rs191790209	1	40125467	T	C	0.6298	0.0084	5.10E-07
rs145127946	1	203746507	A	G	-0.1747	0.037	1.22E-06
rs10495032	1	216837226	T	G	-0.1557	0.0383	9.77E-07
rs536853985	2	1715685	C	G	1.0018	0.9979	8.85E-07
rs1260326	2	27730940	T	C	0.1323	0.3977	1.03E-28
rs7610704	3	11689679	T	C	-0.0568	0.4644	2.21E-06
rs185983693	3	80482685	T	C	0.3206	0.9895	1.83E-06
rs139290229	4	68860848	A	G	0.4402	0.9878	3.27E-06
rs146695032	5	16215858	A	T	-0.6864	0.9947	1.19E-06
rs4700382	5	55882435	T	C	-0.063	0.2698	3.62E-06
rs60277384	5	84356011	T	C	-0.0881	0.8922	3.54E-06
rs116778481	6	14709576	T	C	-1.2617	0.0017	2.18E-06
rs13229619	7	73030175	A	G	-0.1609	0.1297	6.13E-20
rs7012637	8	9173209	A	G	-0.0605	0.4812	4.61E-07
rs80354499	8	60950348	T	C	-0.3503	0.0133	5.40E-07
rs188758663	10	127847758	A	G	-0.3801	0.0104	3.88E-07
rs12290350	11	76480255	T	C	-0.0689	0.2168	2.02E-06
rs2429473	12	47198899	A	C	-0.0812	0.795	3.32E-08
rs535578156	17	43242054	A	G	0.9528	0.0025	4.39E-06
rs532049578	18	41779892	T	C	0.5721	0.0098	4.33E-07
rs190083220	18	43914604	T	G	0.7527	0.9953	2.66E-06
rs72965996	18	72826607	A	G	-0.2215	0.031	6.08E-07
rs838131	19	49260677	A	C	0.1627	0.501	6.15E-36
rs142603673	21	33860969	T	C	-0.2352	0.0232	2.35E-07

Position and CHR: Location of genes and chromosomal information; EA, effect_allele; OA, other_allele; β, Allele effect size; EAF, effect allele frequency.

### 3.2 Results of Mendelian randomization analysis

**Figure 1:**
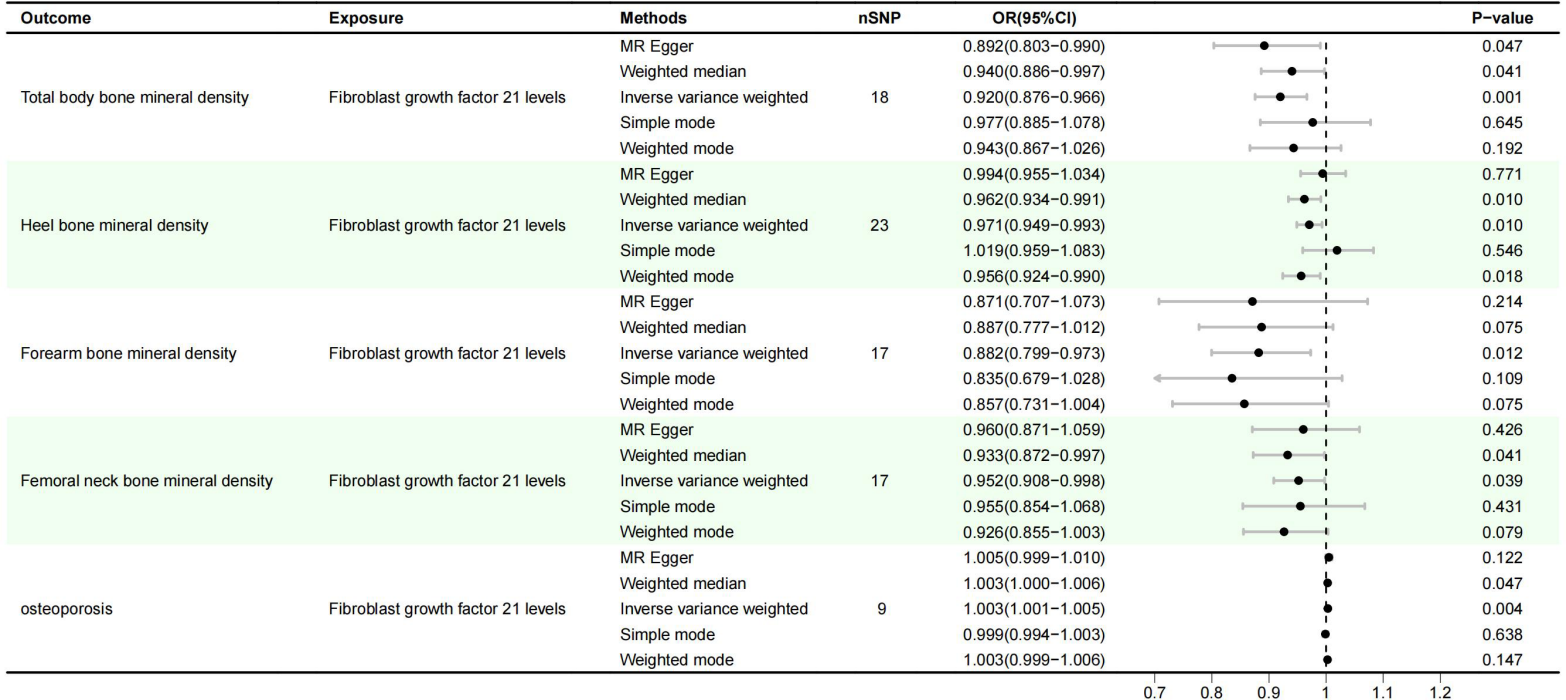
Results of MR Analysis of FGF21 and osteoporosis.

IVW method showed that there was a genetic causal relationship between FGF21 and osteoporosis, and the overexpression of FGF21 increased the risk of osteoporosis (OR=1.003, 95%CI (1.001-1.005), P=0.004). Meanwhile, FGF21 overexpression reduced bone mineral density: Total body bone mineral density (OR=0.920, 95%CI: 0.876-0.966), P=0.001), Heel bone mineral density (OR=0.971, 95%CI (0.949-0.993); P=0.01), Forearm bone mineral density (OR=0.882, 95%CI(0.799-0.973); P=0.012), Femoral neck bone mineral density (OR=0.952, 95%CI(0.908-0.998), P=0.039), (**Figure 1**).

### 3.3 Sensitivity analysis

**Figure 2:**
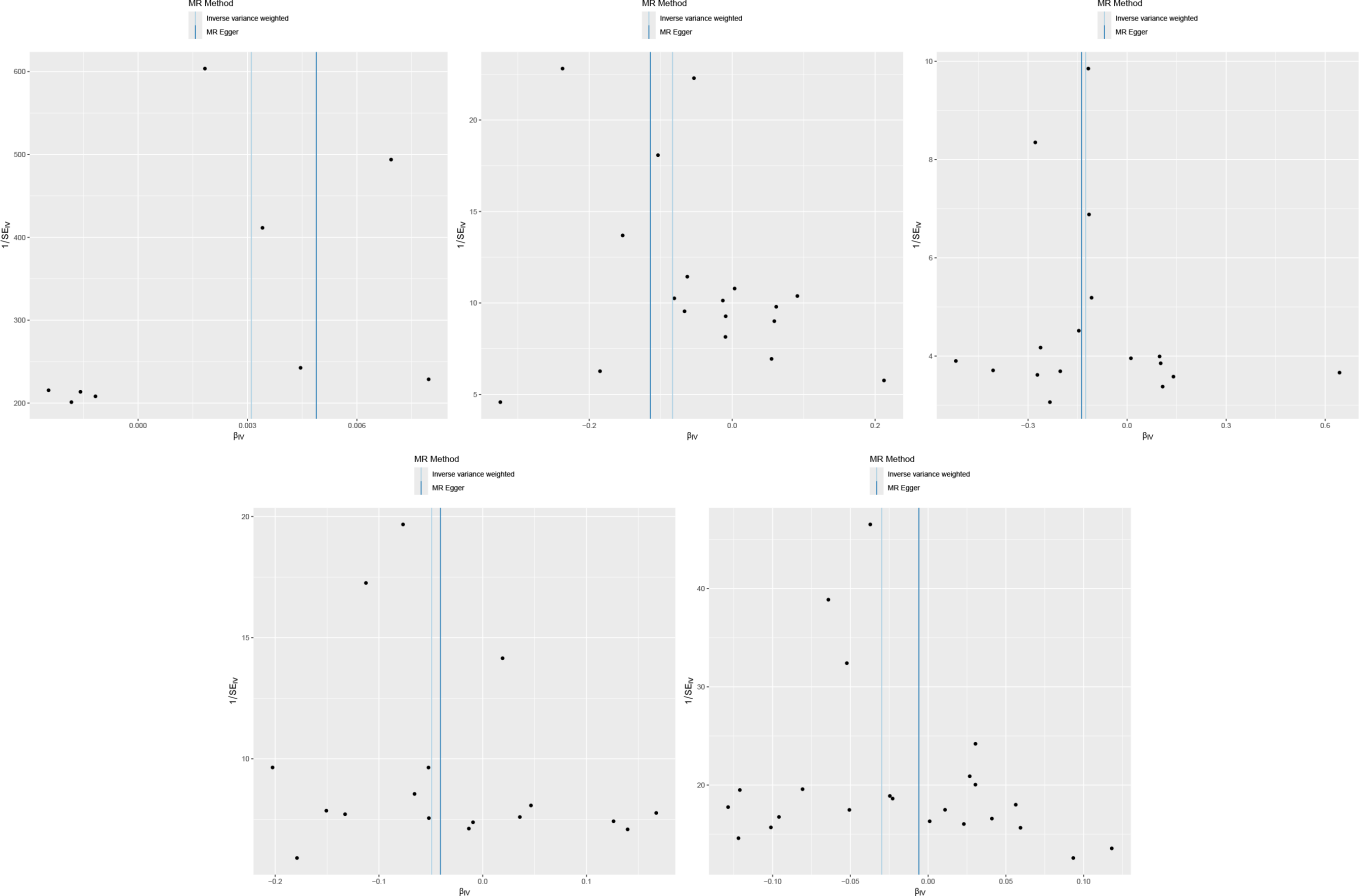
Scatter plot (From left to right, FGF21 and osteoporosis, Total body bone mineral density, Forearm bone mineral density, Femoral neck bone mineral density, Heel bone mineral density).

**Figure 3:**
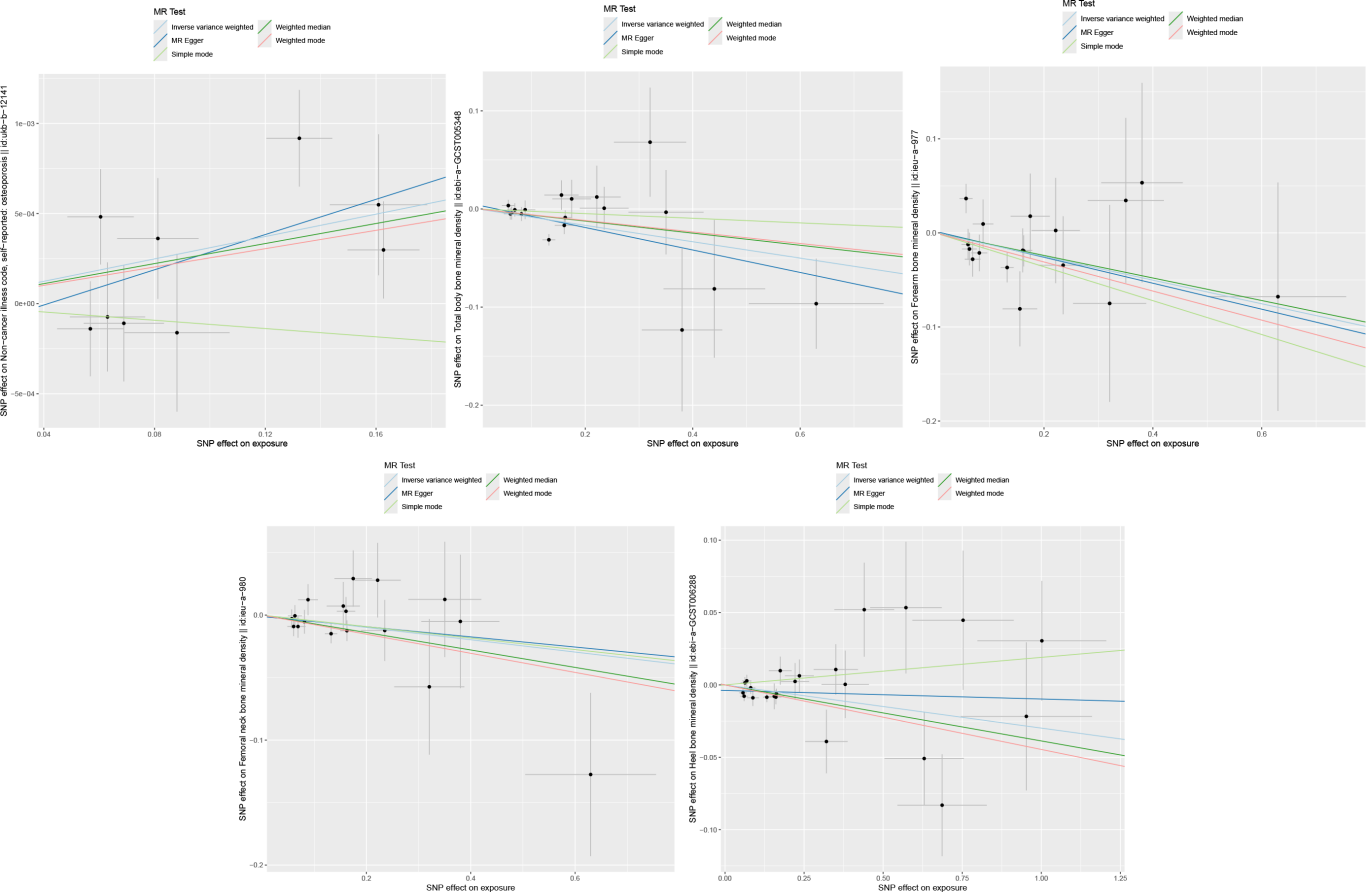
Funnel plot (From left to right, FGF21 and osteoporosis, Total body bone mineral density, Forearm bone mineral density, Femoral neck bone mineral density, Heel bone mineral density).

**Figure 4:**
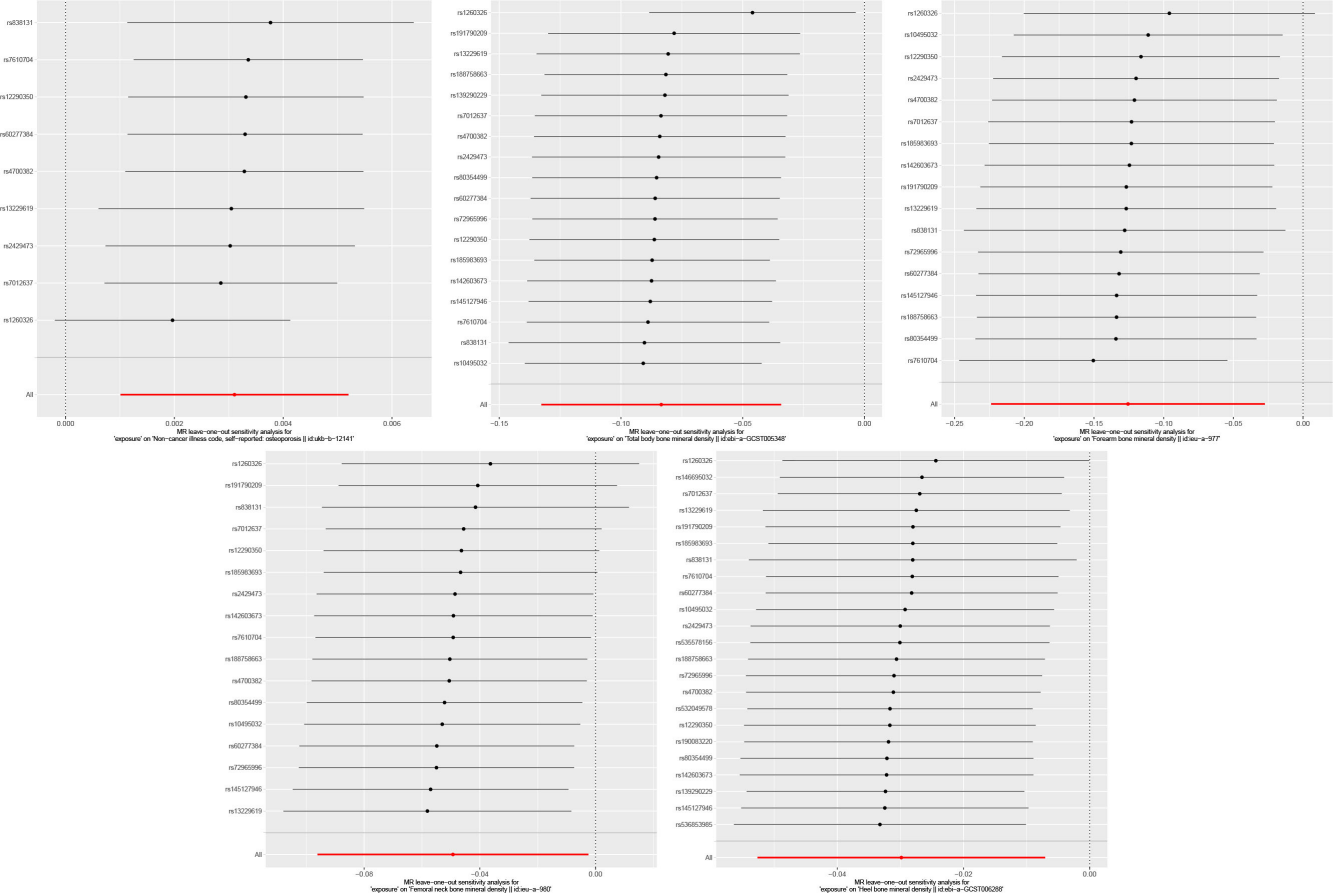
Leave-one-out analysis result (From left to right, FGF21 and osteoporosis, Total body bone mineral density, Forearm bone mineral density, Femoral neck bone mineral density, Heel bone mineral density).

IVW and MR-Egger results showed no heterogeneity between FGF21 and outcome variables (P >0.05), and MR-PRESSO results showed no pleiotropy between FGF21 and outcome variables (P>0.05) (**Figures 2**, **3**). Leave-one-out results showed that excluding each SNPs in turn had no significant effect on the results, which further verified the robustness of the results of this study (**Figure 4**).

### 3.4 Results of mediation analysis

Mediation analysis showed that FGF21 was associated with an increased risk of osteoporosis (OR=1.003, 95%CI (1.001-1.005), P=0.0037). Direct effects indicate that FGF21 has a causal relationship with Type 2 diabetes (OR=0.787, 95%CI (0.646-0.960), P=0.0179) and Basal metabolic rate (OR=0.962, 95%CI(0.935-0.990), P=0.0091). Indirect effect indicated that Type 2 diabetes (OR=0.998, 95%CI(0.997-0.999), P=0.0025) and Basal metabolic rate (OR=0.990, 95%CI(0.987-0.993), P=6.40E-11) also had causal relationship with osteoporosis. In the causal relationship between FGF21 and osteoporosis, the mediating effects of Type 2 diabetes and Basal metabolic rate were 14.96% and 12.21%, respectively (**Table 2**).

**Table 2:** Results of mediation analysis.

Exposure	Mediator	Outcome	Total effect			Direct effect a			Direct effect β			Mediation effect	
			Beta	SE	P	Beta	SE	P	Beta	SE	P	Beta	Proportion
FGF21	Type 2 diabetes	osteoporosis	0.0031	0.0011	0.0037	-0.2393	0.1011	0.0179	-0.0019	0.0006	0.0025	0.0005	14.96%
	Basal metabolic rate					-0.0385	0.0148	0.0091	-0.0099	0.0015	6.40E-11	0.0004	12.21%

## 4 Discussion

The aim of this study was to explore the causal relationship between FGF21 and osteoporosis by two-sample, mediated Mendelian randomization method. MR Analysis showed that FGF21 was negatively correlated with Total body bone mineral density, Heel bone mineral density, Forearm bone mineral density, Femoral neck bone mineral density, and positively correlated with osteoporosis (P<0.05). This suggests FGF21 can decrease the bone mineral density, thus increasing the risk of osteoporosis, which is consistent with the predecessors’ research results (15). Fibroblast growth factor 21 (FGF21) is a protein mainly secreted by the liver and regulated by a variety of physiological conditions and factors (16). Prolonged starvation, overnutrition, and glucagon all upregulated FGF21 expression (17–19), while insulin may inhibit FGF21 expression in the liver (20). In recent years, the relationship between FGF21 and osteoporosis has been continuously explored, and studies have shown that FGF21 is closely related to bone resorption and bone formation. Xu found a negative correlation between serum FGF21 content and PINP in patients with diabetic nephropathy through a cross-sectional study, suggesting that FGF21 may have a certain negative regulatory effect on bone formation (21). Hao collected and analyzed clinical data of more than 700 subjects and found that plasma FGF21 levels were negatively correlated with femoral neck BMD and hip Ward’s triangle BMD (22). Interestingly, FGF21 variant (rs490942) was significantly associated with increased hip Ward triangle BMD in the total cohort and the female cohort, and increased neck BMD in the female cohort. Zhou found that FGF21 levels in the liver and plasma were significantly increased in the osteoporosis rat model after ovariectomy (23). And Wei found FGF21 transgenic mice and FGF21 knockout mice were characterized by low bone mass and bone mass phenotypes (24). Some scholars believe that FGF21 can mediate and enhance the activity of peroxisome proliferator-activated receptorγ(PPAR-γ), thereby inhibiting the formation of osteoblasts and stimulating the adipogenesis of bone marrow mesenchymal stem cells, leading to increased bone fragility (25). Other studies have shown that FGF21 can promote the secretion of hepatic hormone, which binds to osteoclast precursors and promotes osteoclast differentiation, thereby aggravating osteoporosis (26). In addition, Zhou suggested that FGF-21 may mediate osteoclast bone resorption through RANKL/RANK/NFATc1 signaling pathway (23). Although previous studies have proposed the mechanism of FGF21 regulating bone homeostasis from both direct and indirect aspects, more studies are still needed for further investigation.

The results of mediating MR Analysis showed that the causal relationship between FGF21 and osteoporosis was mediated by Type 2 diabetes and Basal metabolic rate, and the proportion of mediating effect was 14.96% and 12.21%, respectively. As an endocrine protein, FGF-21 can regulate liver secretion, glucose and lipid metabolism and other processes. FGF-21 has the ability to inhibit glucagon receptors and improve insulin resistance. It plays an important role in the process of ketogenesis, gluconeogenesis and insulin synthesis, and is a potential therapeutic agent for metabolic diseases such as diabetes (27). In addition, FGF21 also has a certain therapeutic effect on diabetic neuropathy by reducing neuroinflammation and oxidative stress, and enhancing the protection of neuronal mitochondria, thereby alleviating diabetic neurodegeneration (28). Studies have shown that FGF-21 can reduce cell death induced by oxidative stress and autophagy, which is beneficial to remyelination and nerve regeneration after peripheral nerve injury and plays an important role in diabetes (29).Cheng’s study showed that type II diabetes had a protective effect on osteoporosis, and for each additional case of type II diabetes, the incidence of osteoporosis decreased by 0.15% (30). This indicates that type 2 diabetes and osteoporosis are reverse causation, consistent with my results. The relationship between FGF21 and basal metabolic rate has not been reported at present, which may be related to the reduced risk of type 2 diabetes caused by overexpression of FGF21, and more studies are needed to clarify it in the future.

Interestingly, Hao’s study showed that the correlation between FGF21 levels and Neck-BMD was significant, but the relationship was not significant when grouped by gender. At the same time, the correlation between FGF21 and Ward’s BMD was significant in women, while the P-value of men was close to the significance threshold, indicating that men and women were equally prone to be affected by FGF21, and there was no sex specificity (22).However, Lee’s study showed no significant correlation between FGF21 and total BMD and spinal BMD in men. But there was a significant positive correlation in women (31). Whether the effects of FGF21 on bone mineral density and osteoporosis are influenced by gender is still controversial, and more studies are needed to clarify.

## 5 Conclusions

Our study suggests that the increased expression of FGF21 may lead to the decrease of bone mineral density and increase the risk of osteoporosis, while type 2 diabetes and basal metabolic rate may play a mediating role in the relationship between FGF21 and osteoporosis. This study can provide a reference for analyzing the potential mechanism of osteoporosis and is of great significance for the prevention and treatment of osteoporosis.

## Data Availability

The original contributions presented in the study are included in the article/Supplementary Material. Further inquiries can be directed to the corresponding authors.
